# Efficiency Enhancement of Perovskite Solar Cells with Plasmonic Nanoparticles: A Simulation Study

**DOI:** 10.3390/ma11091626

**Published:** 2018-09-05

**Authors:** Ali Hajjiah, Ishac Kandas, Nader Shehata

**Affiliations:** 1 Electrical Engineering Department, College of Engineering and Petroleum, Kuwait University, Safat 13113, Kuwait; 2 Department of Engineering Mathematics and Physics, Faculty of Engineering, Alexandria University, Alexandria 21544, Egypt; ishac@vt.edu (I.K.); n.shehata@kcst.edu.kw (N.S.); 3 Center of Smart Nanotechnology and Photonics (CSNP), SmartCI Research Center, Alexandria University, Alexandria 21544, Egypt; 4 Kuwait College of Science and Technology (KCST), Doha Spur Rd., Safat 13113, Kuwait; 5 USTAR Bioinnovation Center, Faculty of Science, Utah State University, Logan, UT 84341, USA

**Keywords:** Perovskites, solar cell, plasmonic nanoparticles, short circuit current, quantum efficiency

## Abstract

Recently, hybrid organic-inorganic perovskites have been extensively studied due to their promising optical properties with relatively low-cost and simple processing. However, the perovskite solar cells have some low optical absorption in the visible spectrum, especially around the red region. In this paper, an improvement of perovskite solar cell efficiency is studied via simulations through adding plasmonic nanoparticles (NPs) at the rear side of the solar cell. The plasmonic resonance wavelength is selected to be very close to the spectrum range of lower absorption of the perovskite: around 600 nm. Both gold and silver nanoparticles (Au and Ag NPs) are selected to introduce the plasmonic effect with diameters above 40 nm, to get an overlap between the plasmonic resonance spectrum and the requested lower absorption spectrum of the perovskite layer. Simulations show the increase in the short circuit current density (*J_sc_*) as a result of adding Au and Ag NPs, respectively. Enhancement in *J_sc_* is observed as the diameter of both Au and Ag NPs is increased beyond 40 nm. Furthermore, there is a slight increase in the reflection loss as the thickness of the plasmonic nanoparticles at the rear side of the solar cell is increased. A significant decrease in the current loss due to transmission is achieved as the size of the nanoparticles increases. As a comparison, slightly higher enhancement in external quantum efficiency (EQE) can be achieved in case of adding Ag NPs rather than Au NPs.

## 1. Introduction

One of the hottest topics in materials science in the past few years has been hybrid organic-inorganic perovskites due to their superb properties in optoelectronic applications. These organo-metal halide materials have emerged as an excellent absorber material for thin-film photovoltaics with spectacular achievements in power conversion efficiencies that compete with silicon and other established thin-film technologies (i.e., CdTe and CIGS). The power conversion efficiency (PCE) of perovskite based solar cells has increased from 3.8% upon its inception in 2009 to a certified 22.1% in early 2016 [1,2]. The material possesses the ABX3 crystal structure, where A is a small organic cation, B is a cationic group 14 metal, and X is a halide anion. The most commonly used perovskite semiconductor material in solar cells is methylammonium-lead (II)-iodide with the chemical formula CH_3_NH_3_PbI_3_ (MAPbI_3_), owing to its excellent material properties for photovoltaic applications. MAPbI_3_ is an inorganic-organic hybrid perovskite that forms a tetragonal crystal structure and is compatible with both solution processing [3] and evaporation techniques [4,5]. This material is a direct bandgap semiconductor [6] with a bandgap around 1.6 eV and a large open circuit voltage (Voc) of 1.07 V [4], only 0.53 V less than the perovskite bandgap potential (Eg/q) [7]. The bandgap of the MAPbI_3_ perovskite (1.6 eV) can be continuously tuned up to 2.25 eV by substituting Br for I to make MAPb(I_1-x_Br_x_)_3_ [8], which makes perovskite solar cells especially attractive for tandem applications. Furthermore, it is also an intrinsic material with high carrier mobilities [9], high absorption coefficient [10,11], shallow defect levels [12], and a long charge-carrier diffusion length [13,14,15], which are important metrics for highly performing solar cells.

One method for achieving light trapping in thin film solar cells is the use of metallic NPs [16,17,18]. Metallic NPs exhibits the phenomenon of surface plasmon resonance when illuminated with light of suitable frequency [19]. Metallic NPs show potential for enhancing light absorption and photocurrent, therefore, plasmonic resonances in metal NPs have attracted the attention in sensors and other applications such as solar cells [20,21,22]. Plasmonic structure can be integrated with solar cell in many ways [23]. Metal NPs can be deposited on the front surface of the solar cell. Also, they can be embedded inside the cell [24]. However, it was found that locating the particles on the rear side of the absorber layer is more effective in enhancing photocurrent [25,26]. From literature, metal NPs of different size, shape, and composition were used as absorption enhancers in methylammonium lead iodide perovskite solar cells. The absorption enhancement is the key point to reduce the thickness of the perovskite solar cell. Integration plasmonic gold nanostars (Au NSs) into mesoporous TiO_2_ photoelectrodes for perovskite solar cells (PSCs) increased the efficiency from 15.19 up to 17.72% [27,28]. Also, size has been shown to play a pivotal role in performance enhancement. Previous work has systemically screened different AuNPs sizes in photoelectrodes to find the champion devices contained 8 nm plasmonic Au NPs [29]. Incorporation of Au NPs into titanium dioxide (TiO_2_) photoelectrodes showed 20% improvement in average. The refractive index of metal NPs is complex. The permittivity is the square of the refractive index and consequently it is a complex quantity. In optics, the permittivity depends strongly on the frequency. Optical properties of NPs are different from the bulk specimen [30]. Noble NPs can resonate with light, which gives it a great importance. Localized surface plasmons (LSP) can be excited and cause resonance with the incident frequency under certain conditions. The resonant frequency is strongly affected by NPs size, nanoparticle shape, and surrounding medium. Near field enhancement can be exploited in many applications such as solar cell applications [31,32,33,34]. 

In this paper, we focus on the improvement of perovskite solar cell through the addition of plasmonic NPs using a simulation study. The perovskite solar cells may have some lower optical absorption in the visible spectrum around the red region. Therefore, our contribution is to prove the concept that the overall efficiency can be enhanced through adding metallic NPs whose plasmonic resonance wavelength is close to the spectrum range of lower absorption of the perovskite. In more details, Au and Ag NPs are selected to introduce the plasmonic effect with diameters above 40 nm, to get an overlap between the plasmonic resonance spectrum and the lower absorption spectrum of the perovskite layer around 600 nm wavelength. Therefore, this coupling can enhance the quantum efficiency in this spectrum region. In this work, both Au and Ag NPs are selected to be added at the rear-side as an additional layer of perovskite solar cell. Simulated optical properties and quantum efficiency calculations are presented along with a comparison of the impact of both Au and Ag additives.

## 2. Literature Background 

**Figure 1:**
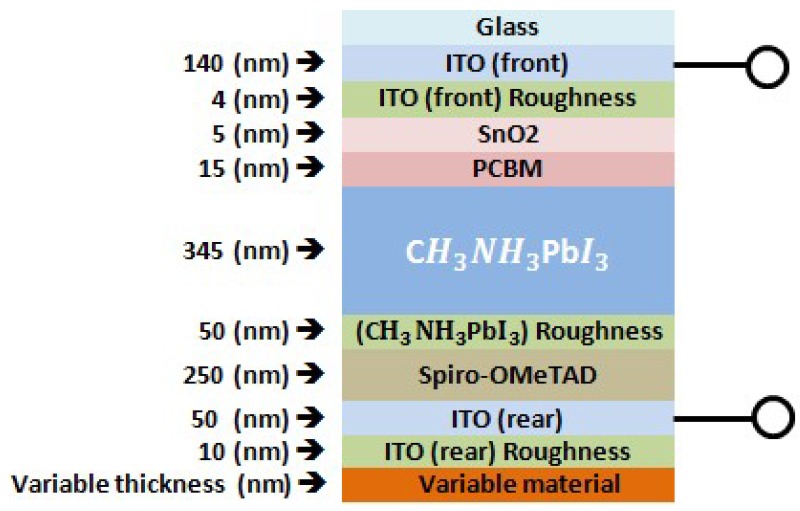
Schematic model of the semi-transparent n-i-p CH_3_NH_3_PbI_3_ perovskite solar cells applied in the transfer-matrix-based optical simulations. The variable material layer is where gold and silver nanoparticles (Au and Ag NPs) are included in our simulations. Roughness is simulated as an effective medium of the adjacent media using the Bruggeman effective medium approximation.

Our targeted device in this work is the regular structure of n-i-p semi-transparent CH_3_NH_3_PbI_3_ perovskite solar cell (area = 0.1–1 cm^2^) with an architecture of glass/ITO-front/SnO_2_/PCBM/CH_3_NH_3_PbI_3_/spiro-OMeTAD/ITO-rear. A schematic model of the semi-transparent perovskite solar cell used in our transfer-matrix-based optical simulations can be seen in Figure 1.

**Figure 2:**
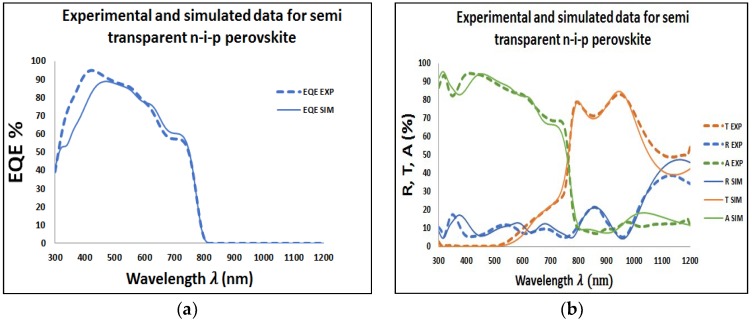
(**a**) Shows measured and simulated external quantum efficiency (EQE)% and (**b**) shows measured and simulated percentages of reflectance, transmittance, and absorbance spectra of semitransparent CH_3_NH_3_PbI_3_ solar cell with the architecture glass/ITO-front/ITO-front-roughness/SnO_2_/PCBM/CH_3_NH_3_PbI_3_/CH_3_NH_3_PbI_3_-roughness/spiro-OMeTAD/ITO-rear/ITO-rear-roughness. The dotted line represents measurements on the actual device and solid line represents transfer-matrix-based simulations.

To investigate the effect of both Au and Ag NPs on the performance of the perovskite solar cell, the Au and Ag NPs were applied at the rear side of the perovskite solar cell with diameter above 40 nm. Then, we can obtain plasmonic resonance wavelength above 550 nm, which can compensate the external quantum efficiency losses in perovskite in this range of spectrum. Then, the Transfer-Matrix-Based Optical Simulation Method (TMM) was used in our investigation. This method allows modeling of the optical properties of thin-film layer stacks by solving Maxwell’s equations at each interface through using the complex refractive index and layer thicknesses of all relevant materials as input [35,36,37,38]. Reflectance (R), Transmittance (T), Absorbance (A), and EQE spectra of the fabricated perovskite solar cell were measured in order to calibrate and underline the accuracy of our optical simulations. More information on the calibration of the TMM optical simulator and the details of the perovskite solar cell fabrication process can be reviewed from our references [39,40]. Surface roughness is considered as an effective medium according to the Bruggeman effective medium approximation (BEMA) [41]. Therefore, in our simulations, interface roughness is simulated using a BEMA layer consisting of a mixture of the optical constants for the adjacent media. The accuracy of our optical simulation is confirmed by showing excellent agreement with experimental data, as shown in Figure 2a,b. However, small offsets between experimental and simulation data for long wavelengths in our transmission and reflection plots can be explained by absorption and/or scattering in the substrate. 

The model used to describe the permittivity of gold and silver is Drude-critical points model. The model can be given in Equation (1) as follows [42]

(1) $$\varepsilon \left(\omega \right)=\varepsilon_{\infty }-\frac{\omega_{D}^{2}}{\omega^{2}+i\gamma_{NP}\omega }+\overset{2}{\underset{p=1}{\sum }}A_{p}\Omega_{p}\left(\frac{e^{i\phi_{p}}}{\Omega_{p}-\omega -i\Gamma_{p}}+\frac{e^{-i\phi_{p}}}{\Omega_{p}+\omega +i\Gamma_{p}}\right)$$

where $\varepsilon_{\infty }$ is the permittivity due to interband transitions, $\omega_{D}$ is the plasma frequency, $\gamma_{NP}$ is the damping constant. $A_{p}$, $\Omega_{p}$, $\phi_{p}$, and $\Gamma_{p}$ are constants and are summarized in Table 1 for gold and silver. The experimental data was taken from reference [43]. The model is valid for wavelengths in the range between 200 nm and 1000 nm [42]. The damping constant, $\gamma_{NP}$ counts for absorption loss is size dependent and given by Equation (2) [44,45,46]

(2) $$\gamma_{NP}=\gamma +\frac{Cv_{F}}{R}$$

where γ equals 1.0805 × 10^14^ rad/s and 4.5841 × 10^13^ rad/s for gold and silver respectively. The constant C is considered 1 for both of them [46], $v_{F}$ is the Fermi velocity and equals 1.39 × 10^6^ m/s and 1.38 × 10^6^ m/s for gold and silver, respectively, and R is the radius of the spherical NPs [45,46]. The refractive index is complex and simply expressed as follows in Equation (3)

(3) $$n=\sqrt{\varepsilon \left(\omega \right)}=n_{r}+ik$$

The real and imaginary parts were calculated for both Au and Ag to be used in the simulation part. To calculate the absorption, scattering, and extinction cross sections of noble NPs, Mie theory can be used. Mie theory is using Maxwell equations to calculate the fields in the vicinity of the nanoparticle. Equation (4) shows the cross sections which can be can be given as [47]

(4a) $$\sigma_{ext}=\frac{2\pi }{{\left|k\right|}^{2}}\overset{\infty }{\underset{n=1}{\sum }}\left(2n+1\right)Re\left[a_{n}+b_{n}\right]$$

(4b) $$\sigma_{sca}=\frac{2\pi }{{\left|k\right|}^{2}}\overset{\infty }{\underset{n=1}{\sum }}\left(2n+1\right)\left[{\left|a_{n}\right|}^{2}+{\left|b_{n}\right|}^{2}\right]$$

(4c) $$\sigma_{abs}=\sigma_{ext}-\sigma_{sca}$$

where $\sigma_{ext}$, $\sigma_{sca}$, and $\sigma_{abs}$ are the extinction, scattering, and absorption cross sections, respectively, *n* is the multipole order, *k* is the wave number. Equation (5) shows the Mie coefficients $a_{n}$ and $b_{n}$, which are given by [47]

(5a) $$a_{n}=\frac{m\psi_{n}\left(mx\right)\psi_{n}^{\prime }\left(x\right)-\psi_{n}\left(x\right)\psi_{n}^{\prime }\left(mx\right)}{m\psi_{n}\left(mx\right)\eta_{n}^{\prime }\left(x\right)-\eta_{n}\left(x\right)\psi_{n}^{\prime }\left(mx\right)}$$

(5b) $$b_{n}=\frac{\psi_{n}\left(mx\right)\psi_{n}^{\prime }\left(x\right)-m\psi_{n}\left(x\right)\psi_{n}^{\prime }\left(mx\right)}{\psi_{n}\left(mx\right)\eta_{n}^{\prime }\left(x\right)-m\eta_{n}\left(x\right)\psi_{n}^{\prime }\left(mx\right)}$$

where $\psi_{n}$, and $\eta_{n}$ are Riccarti –Bessel functions, while $\psi_{n}^{\prime }$, and $\eta_{n}^{\prime }$ are their derivatives.m is the ration of the complex refractive indices of the nanoparticle and the suttrounding medium. *x* is given by 2πR/λ where λ is the wavelength of the incident light.

## 3. Simulation Procedure

In order to investigate the effect of Au and Ag NPs on the performance of the perovskite solar cell, optical loss analysis was considered by varying the thickness of both Au and Ag films at the rear side of the perovskite solar cell in our TMM simulations and calculating both internal current densities losses (*J_short_*, *J_medium_*, *J_long_*), the external losses (*J_escape-back_*, *J_reflection_*), the average transmittance (800–1200 nm), and the *J_sc_* in our perovskite solar cell. Here, we assume one homogeneous layer of NPs on the perovskite cell. Therefore, the thickness of the plasmonic layer is the same as the diameter of NPs. According to the limitations of the used coding we have assumed that Au and Ag NPs or even though the Au and Ag planar film is deposited as one layer over the perovskite with negligible grain boundaries problems that can be found in the real design.

The internal current losses are calculated using Equation (6) by integrating the area between the EQE and the absorbance curves over the AM1.5G solar spectrum for different wavelength regions corresponding to short (λ = 300–450 nm), medium (λ = 450–700 nm), and long (λ = 700–1000 nm) internal current losses. For the external current losses, however, we focused on both losses of *J_escape,back_* and *J_reflection_*. Regarding the *J_escape-back_* external loss, the back side of the semi-transparent perovskite cell was considered where part of the long wavelength light is lost by transmission through the semi-transparent ITO-rear through the nanoparticle material. This external loss is calculated by integrating the transmission curve of the cell according to Equation (7). Moreover, at the device front, part of the light is lost due to external reflection. These reflection losses (*J_reflection_*) are calculated by integrating the reflection spectrum of the front side of the cell according to Equation (8). Then, by using TMM method and Equation (9), *J_sc_* for each specific nanoparticle thickness can be expressed as shown [48].

(6) $$J_{internal-loss}=\frac{q}{hc}\int_{\lambda_{1}}^{\lambda_{2}}\lambda \cdot \Phi \left(\lambda \right)\cdot \left[A_{cell}\left(\lambda \right)-EQE\left(\lambda \right)\right]\cdot d\lambda$$

(7) $$J_{escape-back\;external}=\frac{q}{hc}\int_{500}^{1000}\lambda \cdot \Phi \left(\lambda \right)\cdot T_{cell}\left(\lambda \right)\cdot d\lambda$$

(8) $$J_{reflection}=\frac{q}{hc}\int_{\lambda 1}^{\lambda 2}\lambda \cdot \Phi \left(\lambda \right)\cdot R\left(\lambda \right)d\lambda$$

(9) $$J_{sc}=\frac{q}{hc}\int_{300\mathrm{nm}}^{1000\mathrm{nm}}\lambda \cdot \Phi \left(\lambda \right)\cdot EQE\left(\lambda \right)d\lambda$$

where *q* is the elementary charge, *h* is Planck’s constant, *c* is the speed of the light, *λ* is the wavelength, *Φ(λ)* is the AM1.5G solar spectrum, *T_cell_* is the transmission of the cell, *R* is the reflection of the cell.

## 4. Results & Discussions

### 4.1. Plasmonic Resonances of Au and Ag NPs

**Figure 3:**
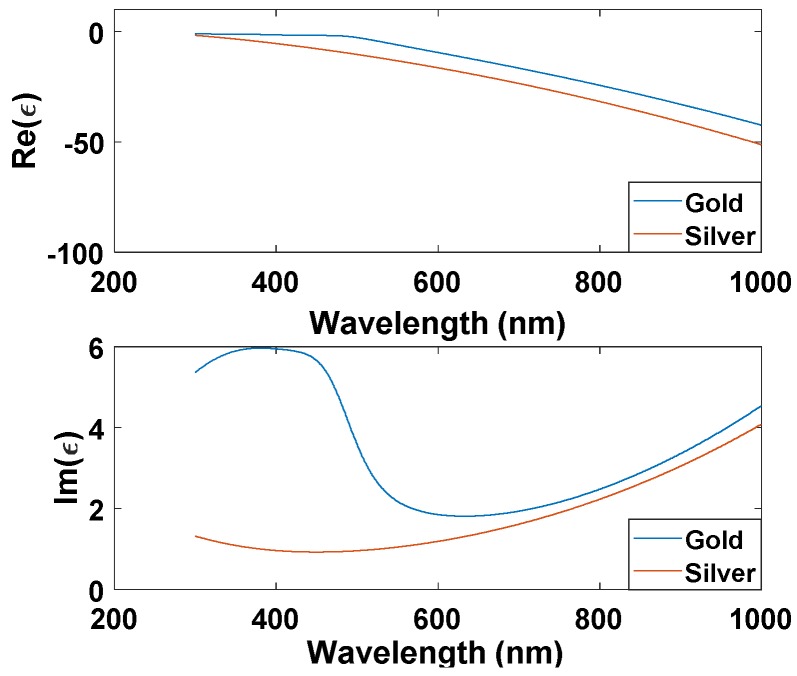
Real and imaginary parts of Au and Ag permittivity.

The real and imaginary parts of Au and Ag NPs are shown in Figure 3. The radius of Ag or Au NPs is 50 nm. All the used parameters are summarized in Table below [36]. As noticed, the real part of Au and Ag permittivity is very close and negative for a wide range of wavelengths. The negative real refractive index is responsible for the appearance of resonance wavelength. The imaginary part is responsible for the absorption loss. As seen, the silver loss is less than the gold loss.

**Figure 4:**
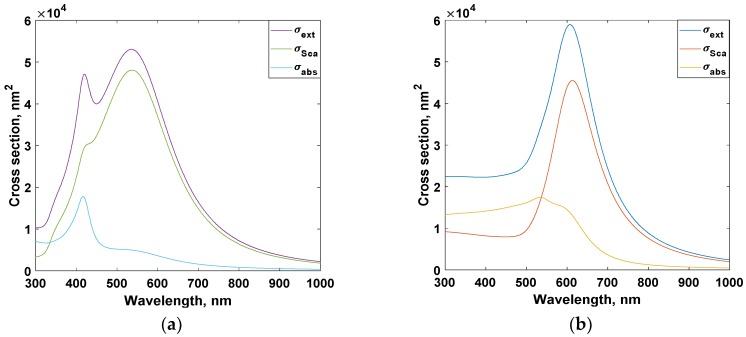
Cross sections of (**a**) Au, and (**b**) Ag plasmonic nanoparticles (NPs).

In Figure 4, the cross sections were simulated for particles of radius 50 nm. The surrounding medium has refractive index of value 1.5. For silver case, the cross section spectrum is much wide and may contain more than one peak. It is worthy to mention that, the resonance wavelength is size dependent. As the nanoparticle size increases, red shift occurs for Au and Ag cross sections. 

### 4.2. Impact of Ag NPs on Perovskite Cells

**Figure 5:**
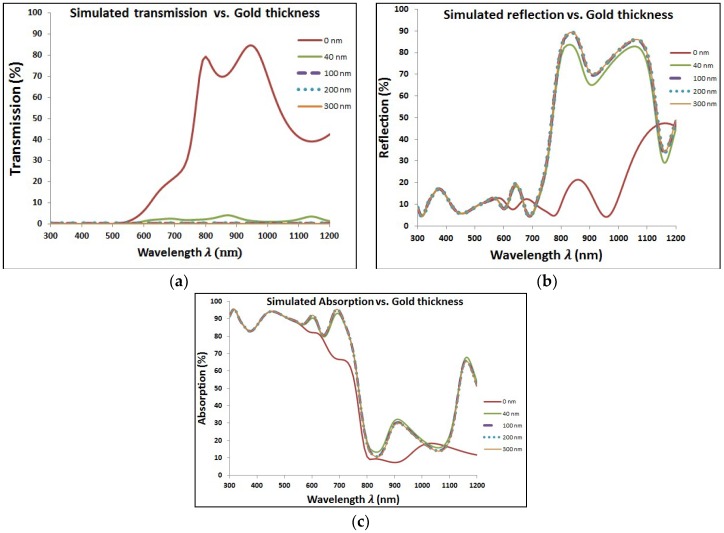
Optical characteristics for perovskite cell with different thickness of gold layer. These characteristics are (**a**) Transmission, (**b**) Reflection, and (**c**) Absorption.

**Figure 6:**
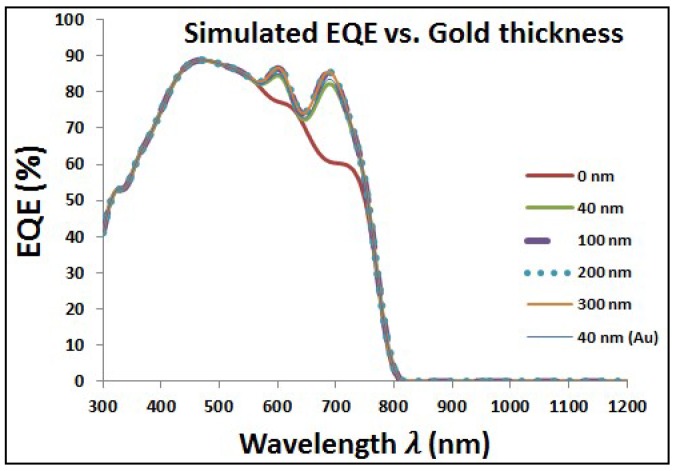
Enhancement of external quantum efficiency (EQE) due to added gold nanoparticles (Au NPs) in the wavelength range close to plasmonic resonance of gold.

Figure 5 shows the optical characteristics of perovskite cell with added layer of Au NPs at different diameters. It can be noticed that the transmission is tremendously decreased with increasing the size of added Au NPs. However, the corresponding reflection and absorption have higher values with added Au NPs especially at wavelength around the plasmonic resonance wavelength. The resonance wavelength is varying from approximately ~550 to ~600 nm when the particle diameter is changing from 40 to 300 nm, respectively, for surrounding medium of refractive index of 1.5. Then, Figure 6 shows the improvement of EQE at wavelength range close to the plasmonic resonance frequency. The EQE enhancement can be explained due to the impact of reflected photoelectrons, which are trapped according to the added layer of plasmonic NPs, beside the optical enhancement of absorption due to plasmonic resonance of the added layer. There is a clear enhancement of EQE with lowest select size of Au NPs. By increasing Au NPs size, the EQE shows slight improvement with a saturation behavior at relatively higher NPs size up to 300 nm in diameter. 

In Table 2, we compare the internal losses, external losses, and the corresponding *J_sc_* of our solar cell with uniform NPs radii at different sizes to show its effect on our simulated perovskite solar cell. Simulations show a 1.24 mA/cm^2^ increase in the *J_sc_* as a result of adding 40 nm Au NPs at the rear side of our perovskite solar cell. Further enhancement in *J_sc_* is observed as the diameter of the Au NPs is increased beyond 40 nm. However, an insignificant difference in *J_sc_* (≈0.1 mA/cm^2^) is observed when comparing the 40 nm Au NPs with a 40 nm Au planar thin film. The Au planar perovskite solar cell is displayed in order to better assess the performance of our Au NPs proposed solar cell structure. Furthermore, it can also be seen from the table a slight increase in the reflection loss as the thickness of the Au NPs at the rear side of the solar cell is increased. However, with increasing Au NPs thickness at the rear side of the perovskite solar cell, a noticeable decrease in the current loss due to transmission (*J_escape,back_*) can be seen with nearly neglected value at 200 nm size or higher which shows a clear advantage of the added NPs.

### 4.3. Impact of Ag NPs on Perovskite Cells

**Figure 7:**
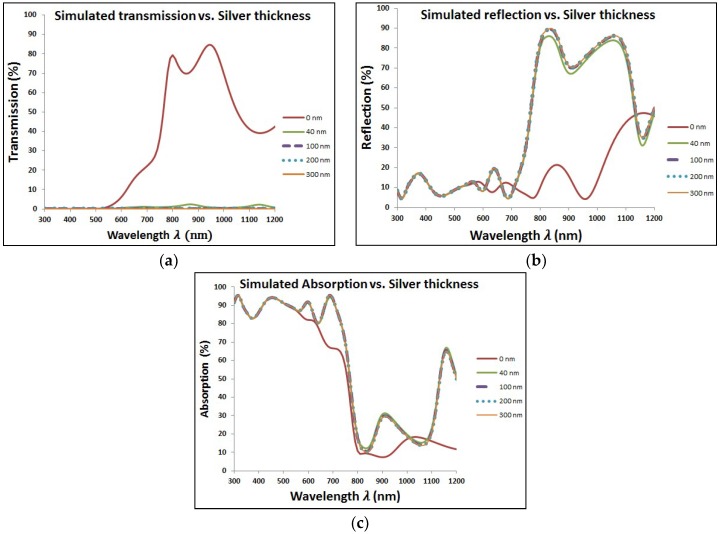
Optical characteristics for perovskite cell with different thicknesses of silver layer.

**Figure 8:**
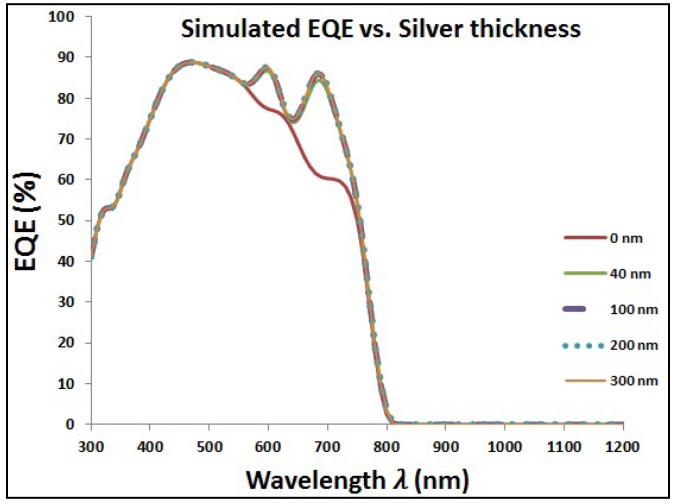
Enhancement of external quantum efficiency (EQE) due to added silver nanoparticles (Ag NPs) in the wavelength range close to plasmonic resonance of silver.

The same behavior of added Au NPs is repeated with another type of plasmonic nanostructure, which is silver. Figure 7 shows the optical characteristics of perovskite cell with added layer of Ag NPs at different diameters. As the case of gold, adding Ag NPs makes the transmission worse, but with improved reflection and absorption, especially at close wavelength-range to the plasmonic resonance of silver. The overall impact is the enhancement of EQE with added Ag NPs as shown in Figure 8. Table 3 shows the results of different types of current losses with different thickness of silver layer. Same behavior is existed as found in Table 1 in the case of Au NPs via enhancement of both reflection and short circuit currents. However, silver is showing higher enhancement in short circuit currents and faster decay of backside escape current with increasing the diameter of Ag NPs compared to gold.

### 4.4. Comparison Between Gold and Silver Effects

**Figure 9:**
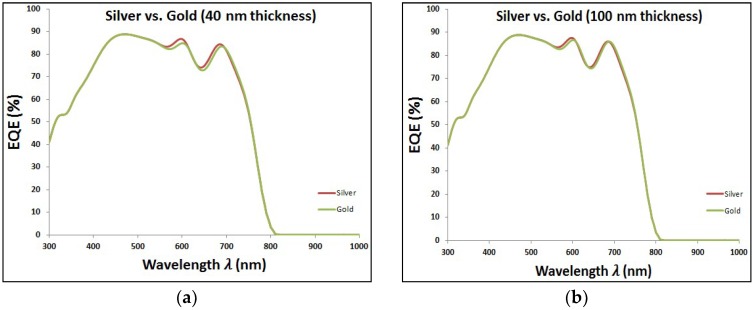

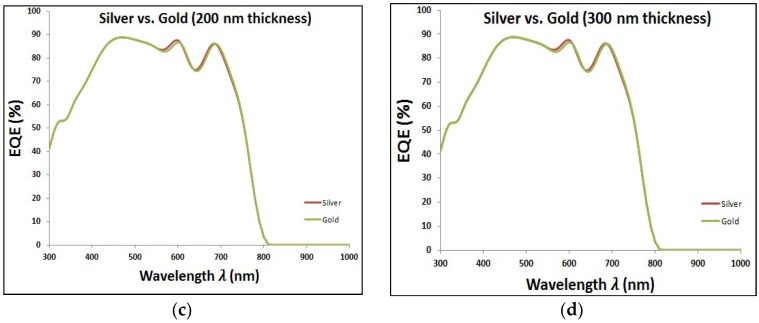
Comparison of external quantum efficiency (EQE) curves at added Au and Ag at different size of nanoparticles (NPs).

**Figure 10:**
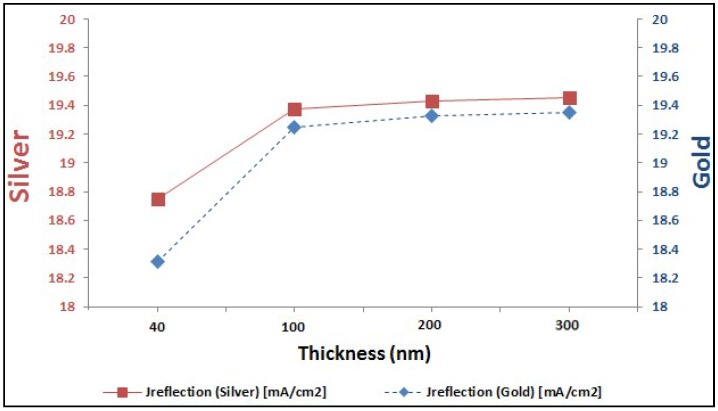
Optical reflection due to Au and Ag at different size of nanoparticles (NPs).

**Figure 11:**
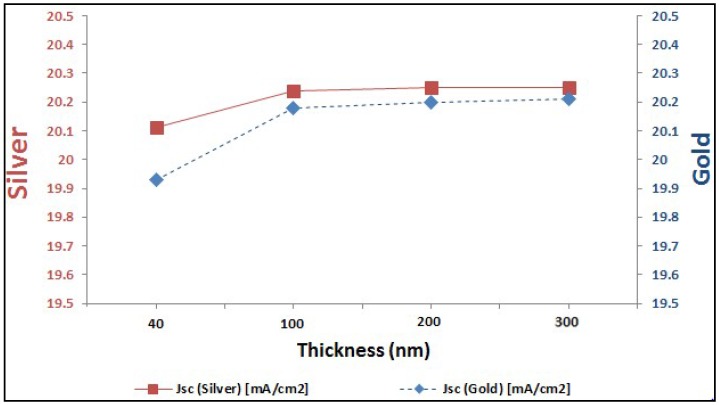
Comparison of *J_sc_* values in the cell according to added Au and Ag at different size of nanoparticles (NPs).

In last two sections, it is proved the enhancement of EQE of perovskite solar cells with added plasmonic NPs. Here, we are going to compare between both added plasmonic Au and Ag NPs. It can be shown from Figure 9 that Ag NPs enhance the EQE of perovskite cell slightly more than the added gold for different thicknesses of plasmonic layer. It can be explained through the better reflection capability that silver can offer compared to gold, as shown in Figure 10. This consequently leads to higher enhancement of short-circuit current in the case of silver compared to gold, as shown in the comparison figure at different layer thicknesses in Figure 11.

## 5. Outlook

To fully benefit from the plasmonic effects of adding Au or Ag NPs at the rear-side of perovskite solar cells, the existing key optical losses need to be identified and addressed. These optical losses can be attributed to: a) overall reflection b) free carrier absorption in ITO electrodes, c) parasitic losses due to absorption in the substrate and diffuse scattering. To minimize the loss due to reflection, anti-reflective coating (ARC) or nanophotonic transparent electrodes can be used to improve the overall Power Conversion Efficiency (PCE) of the solar cell [49]. Furthermore, novel high-mobility Transparent Conducting Oxides (TCOs) such as hydrogenated indium oxide exhibit lower free carrier densities than the commonly used Indium Tin Oxide (ITO), hence offering the chance to minimize the parasitic absorption in ITO electrode at the front side [50,51]. Moreover, to completely mitigate the parasitic losses due to absorption in the substrate and diffuse scattering, ultra smooth CH_3_NH_3_PbI_3_ films and non-absorbing substrates need to be used. More advanced light management concepts, trapping textures, and the optimization of the bandgap of the perovskite bear the potential to lead to very significant further improvements in light harvesting and current generation [52]. 

## 6. Conclusions

In this paper, the effect of adding plasmonic layer of Au and Ag NPs to the rear side of the perovskites solar cell is analytically studied. The resonance wavelength of the plasmonic NPs is adjusted to enhance the optical absorption of the solar cell in the visible range especially around the red wavelength. Both gold and silver lead to a promising enhancement in *J_sc_* when the nanoparticle size becomes larger than 40 nm. Overall EQE enhancement is achieved. EQE improvement is slightly higher in case of adding silver compared to added gold. 

## Figures and Tables

**Table 1:** Parameters used in Drude model.

	$\varepsilon_{\infty }$	$\omega_{D}\;(\mathrm{rad}/s)$	A1	A2	$\phi_{1}$	$\phi_{2}$	$\Omega_{1}\;(\mathrm{rad}/s)$	$\Omega_{2}\;(\mathrm{rad}/s)$	$\Gamma_{1}\;(\mathrm{rad}/s)$	$\Gamma_{2}\;(\mathrm{rad}/s)$
Au	1.1431	1.3202 × 10^16^	0.2669	3.0834	−1.2371	−1.0968	3.8711 × 10^15^	4.1684 × 10^15^	4.4642 × 10^14^	2.3555 × 10^15^
Ag	15.833	1.3861 × 10^16^	1.0171	15.797	−0.9394	1.8087	6.6327 × 10^15^	9.2726 × 1017	1.6666 × 10^15^	2.3716 × 10^17^

**Table 2:** The effect of added gold nanoparticles (Au NPs) of different nanosize on the internal losses, external losses, and the short circuit current for our simulated Perovskite solar cell.

Perovskite Cell Losses (mA/cm^2^)				$J_{sc}(\mathrm{mA}/{\mathrm{cm}}^{2})$
Gold (nm)	$J_{Internal}$	$J_{escape,back}$	$J_{reflection}$	
0	4.15	16.49	6.55	18.69
40 (Au) planar	6.939	0.784	18.7	20.03
40 (Au NPs)	7.45	0.764	18.31	19.93
100 (Au NPs)	7.013	0.009	19.25	20.18
200 (Au NPs)	6.926	0.000	19.33	20.20
300 (Au NPs)	6.899	0.000	19.35	20.21

**Table 3:** The effect of added silver nanoparticles (Ag NPs) of different nanosize on the internal losses, external losses, and the short circuit current for our simulated Perovskite solar cell.

Perovskite Cell Losses (mA/cm^2^)				$J_{sc}(\mathrm{mA}/{\mathrm{cm}}^{2})$
Silver (nm)	$J_{Internal}$	$J_{escape,back}$	$J_{reflection}$	
0	4.15	16.49	6.55	18.69
40	7.182	0.41	18.75	20.11
100	6.846	0.002	19.37	20.24
200	6.778	0.000	19.43	20.25
300	6.756	0.000	19.45	20.25
